# Pain Assessment in Nursing Home Residents Living with Dementia: Current Findings and Concerns

**DOI:** 10.20900/agmr20260007

**Published:** 2026-04-09

**Authors:** Barbara Resnick, Rachel McPherson, Nayeon Kim, Elizabeth Galik, Shijun Zhu

**Affiliations:** Department of Organizational Systems and Adult Health, University of Maryland School of Nursing, 655 West Lombard Street, Baltimore, MD 21201, US

**Keywords:** pain, dementia, nursing home

## Abstract

Prior research has noted that pain has a significant impact on quality of life, behavioral symptoms, and disease progression among older adults living with dementia. There are many factors associated with pain, and challenges to identifying pain. The purpose of this study was to describe current findings related to pain assessment among residents living with dementia in nursing homes and consider if pain is optimally evaluated. This was a descriptive cross-sectional study using baseline data from an ongoing cluster randomized trial testing the implementation of the Pain Management Clinical Practice Guideline in nursing homes. Descriptive data and medication and nonpharmacologic treatments were obtained from the Electronic Health Record. Pain assessments were obtained from the Minimum Data Set and the Pain Assessment in Alzheimer’s Disease was obtained by research evaluators. A total of 156 residents from 7 nursing homes were included. Evidence of pain ranged from 13% (verbal report) to 27% (objective assessment). The majority of assessments by staff used verbal MDS assessments. Only cognition, neurological, and musculoskeletal conditions were associated with pain. The findings from this study reinforce the challenges in measurement of pain among older adults living with dementia, support the possible benefit of using an objective measure of pain, and serve as a reminder to not only evaluate pain but to manage it with nonpharmacologic and pharmacologic interventions.

## INTRODUCTION

Prior research suggests that pain is a complex symptom that incorporates sensory and emotional experiences and is unique for every individual. The prevalence of pain is high among nursing home residents, including those living with dementia, ranging from 24% to 80% depending on the population [1-3]. Due to challenges in assessing and identifying pain among residents with dementia they are often less likely to be described as having pain compared to residents without dementia [3]. This is particularly noted when pain is assessed using the Minimum Data Set (MDS) [4]. The MDS is part of the U.S. federally mandated process for clinical assessment of all residents in Medicare or Medicaid certified nursing homes. This assessment process provides a comprehensive evaluation of each resident’s functional capabilities and health status. The MDS verbal pain assessment requires that the resident recall pain over the past 5 days. This level of recall is not likely to be accurate or reliable among those with moderate to severe cognitive impairment [3]. When pain is not identified residents are at risk for decreased quality of life, increase in behavioral symptoms, and progression of disease or the underlying cause of the pain.

The brain processes pain through medial, lateral and rostral pain networks and the descending pain modulatory system [5]. The medial pain network identifies the unpleasantness of the pain, the lateral pain network focuses on the intensity of the pain, and the rostral pain network leads to the behavioral responses to pain [5]. Changes in the brain throughout the progression of dementia may alter how the individual experiences pain. Despite these changes, individuals living with dementia do have pain. A systematic review [6] of 18 studies using experimentally evoked pain with psychophysical methods (e.g., heat sensitivity, electric-shock and tourniquet induced ischemia) concluded that older adults living with dementia consistently experienced pain and the pain was more unpleasant for them than for individuals who did not have cognitive impairment.

### Factors Associated with Pain Among Older Adults Living with Dementia

Although it may vary by study, there is some evidence that females, those living in rural settings, those with musculoskeletal disorders, neurological disorders, sleep disorders, and anxiety or depression were more likely to have pain than those who were male, living in more urban areas, and not experiencing these comorbidities [3,7]. There is also some evidence to suggest that diabetes and obesity are associated with pain but this has not been comprehensively studied [4].

### Challenges to Assessment of Pain Among Residents Living with Dementia

There are many challenges to identifying pain among residents living with dementia starting with their inability to recall the pain experienced. This is particularly a concern as the resident is asked to recall pain over a 5-day period which is something that those with any level of short-term memory changes (e.g., in ability to recall 1, 2 or 3 items during cognitive testing) will be unable to do. Further the verbal items on the MDS are particularly difficult for individuals with cognitive impairment to respond to as they require not only recall but also quantification of the pain in terms of how much time the individual experienced pain. Another challenge is their inability to verbally express that they are having pain, particularly in later stages of the disease. It is consequently recommended that if an individual cannot express him or herself, observed symptoms of pain should be considered [8]. These include facial expressions, body positioning, resistiveness to care, agitation, decreased engagement, decreased appetite, or physiological changes such as increased heart rate [9,10]. Other challenges include the lack of sensitivity in observation of these symptoms as some may be due to causes other than pain such as normal skin changes (e.g., facial wrinkling) or neurological conditions (e.g., Parkinson’s disease); lack of knowledge among health care providers or family caregivers to identify signs and symptoms of pain; and older adults tendency to consciously or unconsciously manage pain by positioning and not moving to avoid any discomfort [7,11]. Given staff turnover, it is also challenging to accurately evaluate pain when the caregiver does not know the resident and his or her baseline activity and behavior [10,12].

Much has been done to acknowledge that older adults living with dementia do have pain, that there are many factors associated with experiencing pain, and many challenges to identifying pain among these individuals. The purpose of this study was to describe current findings related to pain assessment among residents living with dementia in nursing homes and evaluate if pain is optimally evaluated in these nursing homes. In addition, we explored the factors that were noted to be associated with pain when the different measures were used. Specifically, it was hypothesized that controlling for age, sex, race, and marital status, that cognition, musculoskeletal disease, neurological disease, diabetes, use of pain medications or nonpharmacologic interventions for pain would be associated with verbal MDS assessments, observational MDS assessments, and the Pain Assessment in Advanced Dementia (PAINAD) [13].

## MATERIALS AND METHODS

This was a descriptive, cross-sectional study using baseline data from an ongoing cluster randomized trial testing the implementation of the Pain Management Clinical Practice Guideline in Nursing Homes. The study testing the implementation of the Pain Management Clinical Practice Guideline in Nursing Homes was approved by the University of Maryland Institutional Review Board and has been registered on clinicaltrials.gov (NCT05858996).

### Sample

Residents were recruited from seven nursing homes in Maryland. Eligibility was based on: (1) living in one of the seven nursing homes; (2) being 60 years of age or older; (3) screen positive for dementia based on a recommended process [14] that includes: a score of 0–13 on the Brief Interview of Mental Status (BIMS) [15]; a score of >2 on the AD8 Dementia Screening Interview [16]; a score of 0.5 to 2.0 on the Clinical Dementia Rating Scale (CDR) [17]; and lastly a score of 9 or greater on the Functional Activities Questionnaire (FAQ) [18]; and (4) evidence of pain based on the Minimum Data Set assessment item: How much of the time over the past 5 days have you experienced pain or hurting with eligibility based on the following responses: occasionally, frequently or almost constantly; staff report of pain at the same frequency; or if the resident was receiving nonpharmacological or pharmacological treatment for pain. Exclusion criteria included: (1) those admitted for rehabilitation or short-term subacute care. A list of potentially eligible residents was obtained from a designated staff member and residents were randomly approached, informed about the study and completed the Evaluation to Sign Consent [19] with a research team member. If they passed this evaluation, they could self-consent. If they did not pass the evaluation, the legally authorized representative was contacted to complete the consenting process. Informed consent was obtained from all participants.

### Measures

Descriptive resident data included age, race, sex, ethnicity, marital status, education, total comorbidities were calculated based on a sum total of evidence of the following 13 comorbid conditions based on chart review including: cardiac, vascular, neurological, endocrine, liver, renal, urologic, upper gastrointestinal, lower gastrointestinal, hematopoietic, ear, nose or throat, respiratory, or psychological disease). Comorbidities specifically relevant to pain included evidence of musculoskeletal disease such as osteoarthritis, neurological disease such as a stroke or Parkinson’s disease, and diabetes based on chart review. Cognition was based on the Brief Interview for Mental Status (BIMS) as this is the measure that is routinely used in nursing homes as part of the Minimum Data Set (MDS) [15]. The MDS is a required assessment in Medicare covered nursing homes in the United States and must be done on admission, quarterly or if there is an acute change in condition of the resident (e.g., following a fall, pneumonia, a hospitalization). BIMS is a screening tool for dementia with scores ranging from 0 to 15. Reliability and validity have been established for use with nursing home residents [15]. Based on prior research [10,15] BIMS scores of 0 to 7 are considered to be indicative of moderate to severe cognitive impairment and scores of 8 to 12 are considered to be mild cognitive impairment [18]. The BIMS score and the pain assessments obtained from the MDS were the from the same assessment period and were the most recent scores available from the electronic health record.

Pain was evaluated by nursing staff based on the required and relevant items in the Minimum Data Set (MDS) (Table 1). The assessment was based on a total score was obtained from the items on the observation scale from the MDS with a possible range from 0 to 4 and verbal items which ranged from 0 to a possible high score of 21. When BIMS scores were 8 to 12, it was expected that the staff would evaluate pain using either the verbal or observation MDS items. When scores were 0 to 7 it was expected, as per MDS guidelines, that staff would use the observation measure of pain [10,15]. The BIMS scores and MDS data were obtained from the electronic health record by the research evaluators. The PAINAD was added as a way to determine if use of this observational measure done by research evaluators during some type of activity would be more likely to identify pain among residents living with dementia. The BIMS scores and MDS pain assessments used were the ones from the medical records that were closest in time to when the PAINAD was completed by the research evaluators. In addition, the PAINAD [13] was completed on all participants by the research evaluators at the time of recruitment. The PAINAD is a brief observational measure of pain that evaluates residents for five signs of pain: breathing pattern, negative vocalization, facial expression, body language and consolability. Each item ranges from a score of 0 to 2 and total scores range from 0 (no pain) to 10 (severe pain). Prior testing of the measure when used with older adults provided support for the reliability and validity [20,21]. The evaluators observed the participant for pain during some type of activity. Activities included such things as range of motion, bathing, bed mobility, transferring, repositioning, walking or wheeling oneself (see Table S1). The use of pharmacologic and nonpharmacologic interventions were also collected from the electronic health record at the baseline assessment and included the two weeks prior to baseline. We focused on whether or not treatments were ordered (excluding those order only as needed) and summed the total number of pharmacologic treatments ordered and the total number of nonpharmacologic treatments ordered.

### Data Analysis

Descriptive data was done to describe the sample using SPSS version 29.0 based on means and standard deviations (SDs) for continuous variables and frequencies for dichotomous variables. The pain assessments were all skewed with more than 70% having no pain and so these outcomes were dichotomized such that if there was a score of 1 or greater on any of the measures it was considered evidence of pain. To test the hypothesized relationships, generalized linear mixed models with dichotomous outcomes were used to account for clustering within facilities. STATA was used for these analyses. Separate models were built for each of the three different approaches to pain assessment. The random intercept was included to account for potential clustering within facilities. Fixed effects included demographic variables, cognition, musculoskeletal disease, neurological disease, diabetes, and total pain management dedications. There were only 26 assessments done using the MDS observation measure and this model was run without demographic variables included. A p ≤ 0.05 was used to determine significance. Missing data was limited with pain assessment missing on only 2 (1%) participants and missing at <5% across all other variables. Therefore, no additional missing data analyses were performed. Multicollinearity was evaluated using the variance inflation factor (VIF) and correlation matrix. All variables had a VIF of between 1 and 1.33 with small correlation coefficients (all r’s < 0.3) among them, indicating no evidence of multicollinearity.

## RESULTS

As shown in Figure 1, we assessed 337 residents for eligibility based on chart review across 7 communities. Among these 337 individuals, 156 were enrolled and 181 were not enrolled due mainly to refusals (40 residents refused assent and 44 legally authorized representatives refused to consent), 13 residents did not meet eligibility based on age or not having pain, and 25 residents were not eligible following consent as they did not meet cognitive inclusion criteria. Further 59 residents were not included due to other reasons as shown in Figure 1.

As shown in Tables 2 and 3, the mean age was 79.86 (SD = 9.56) and the majority was female (69%), White (54%), not married (83%), and had at least a high school education (87%). The mean cognitive score based on the BIMS was 5.52 (SD = 4.04) completed by researchers. Participants had a mean of 5.85 (SD = 2.00) comorbidities. A total of 133 (85%) of the residents were evaluated by nursing home staff using the verbal MDS pain assessment questions and the mean pain score was 1.03 (SD=3.03) with only 13% having some pain. A total of 21 (14%) residents were evaluated by staff using the observation items from the MDS with a mean score of .50 (SD = 0.91) and 27% were noted to have pain. Of the 21 individuals evaluated using the observational items in the MDS, 10 (7%) individuals were evaluated using the observation items only and 11 individuals (7%) were evaluated using both observational and verbal items. Among the 88 participants with a BIMS score of 8 to 12, the majority (N = 86, 98%) were evaluated using a verbal assessment. For the 66 individuals with a BIMS score of less than or equal to 7, only 14 (21%) were evaluated using the observation assessment in the MDS. Tables S2 and S3 provide the frequencies for the responses for each of the items on the MDS pain assessments. The PAINAD evaluations done on all participants resulted in a mean score of 0.75 (SD = 1.48), a median score of 0, and 27% having evidence of pain.

Nonpharmacologic interventions ordered are shown in Table S4. The majority of interventions ordered included positioning, ointments and pain patches that did not have pharmacologic agents, physical activity and rehabilitation referrals for exercise. Pharmacologic treatment most commonly included acetaminophen (67%), local treatments such as aspercream or a lidocaine patch (20%), gabapentin (14%), opioids 12%), tramadol (4%), nonsteroid anti-inflammatory agents (2%), pregabalin (2%) and muscle relaxants (1%) (Table S5).

As would be expected, controlling for age, sex, marital status and race, cognition was the only variable associated with verbal assessment of pain based on the MDS measure completed by staff (Table 4). Those with better cognition were 19% more likely to verbally report pain based on the MDS assessment items (OR = 1.19, 95% CI: 1.04, 1.37, p = 0.010). There was no association with any of the variables and the MDS observation measure items. The only variable significantly associated with pain measured using the PAINAD was having musculoskeletal disease. Those with musculoskeletal disease were 5.30 times more likely to report pain based on observation using the PAINAD.

## DISCUSSION

Overall, the participants in this study were noted to have relatively low levels of pain. This low incidence of pain among residents living with dementia is consistent with some prior studies [22,23] although the findings from studies vary based on the measures used, the evaluators (e.g., staff, family), and whether or not the individual was at rest or engaged in activity during the testing. It is possible that this low level of pain is due to the challenges older adults with moderate to severe dementia face in recognizing, reporting or recalling pain. Further pain assessments were limited as we did not have comprehensive data on the exact timing of when the assessment was done (e.g., morning or evening), the activity the resident was doing, environmental factors, exposure to and timing of treatment among other factors.

The majority of the residents with BIMS scores of 0 to 7 were evaluated by the staff using the verbal MDS. We anticipate that verbal assessments were done as these were quicker for staff to do. Further the staff may also have had a lack of appreciation of the challenges these individuals have regarding identification and recall of pain. The greater percentage of individuals identified with pain using the objective measure suggests that it may be useful to include an observation approach to measure pain for those with cognitive impairment (i.e., at least for those scoring 0–7 on the BIMS) [9,10]. Further, it is possible that an observation measure such as the Pain Assessment Checklist for Seniors with Limited Ability to Communicate [24], which has 31 observation items, might help identify a greater percentage of individuals with pain.

The hypothesis proposing that cognition, musculoskeletal disease, neurological disease or diabetes, use of pain medications or nonpharmacologic interventions would be associated with verbal and objective measures of pain was minimally supported in this study. Only cognition and having neurological diseases were significantly associated with the verbal assessment of pain based on the MDS items. Consistently, those with better cognitive ability are more likely to verbally report pain when compared to those with less cognitive ability [10,17,25]. The Long-Term Care Facility Resident Assessment Instrument 3.0 User’s Manual [26] strongly encourages the use of verbal reporting of pain directly from the resident as long as the resident can at least sometimes be understood [27]. This may result in missed findings of pain as those scoring low on the BIMS (0 to 7) are not likely to be able to recall pain over the past 5 days even if they are verbally able to make themselves understood [17,25].

We did not detect a clear relationship between treatment for pain (pharmacologic and nonpharmacologic) and verbal or objective assessments of pain. This may be due to limited information about the amount and timing of drugs or treatments provided. Further, we did not include as needed pain medication and thus the use of medications might actually have been higher. It is possible, however, that pain may not be addressed among these individuals due to concerns about drug side effects or lack of effectiveness of nonpharmacologic approaches among individuals with moderate to severe cognitive impairment or that the pain was resolved. Prior research has also noted that there was no association between treatment and actual pain measured objectively or verbally among older adults living with dementia [28-31]. Further it is possible that the lack of use of pain assessments to guide the treatment of pain may be due to fluctuations in pain assessments done at different points in time. These differences may result in hesitation to initiate treatment. Pain protocols providing care guidelines such as use of clinical practice guidelines have been shown to be effective in improving the relationship between pain assessment and management in some studies [32,33]. All members of the team need to be included in the pain protocol so that there is not over dependence on pharmacologic versus nonpharmacologic interventions [32].

Musculoskeletal disease was associated with observations done during the PAINAD. The PAINAD was assessed during some type of activity and it is possible that the movement of joints during the assessment resulted in responses such as grimacing or resisting care. Having diabetes may not have been associated with pain in these individuals as pain among diabetics is commonly due to neuropathies which only occurs in some diabetic patients.

Differences in pain assessment findings between observation and verbal reporting of pain have previously been noted [10,17,34]. To optimally evaluate pain, a systematic review of 39 studies indicated that health care providers doing the evaluations need to: (1) be aware of pain and pain signs and symptoms among individuals with dementia; (2) suspect/anticipate pain in these individuals; and (3) consider pain mapping which involves a process of trying to identify the triggers of pain and/or the underlying cause of the pain. Alternatively, other types of assessment tools have been recommended such as the use of technology and more comprehensive assessment of facials expressions to identify pain among individuals living with dementia [9] or use of biologic or genetic markers [35].

### Study Strengths and Limitations

A strength of this study was the in-depth extraction of data from the electronic health record by trained research evaluators. Further, although the sample was not large, residents were from seven different communities and there was a high percentage of males and Blacks compared to other nursing home studies. For eligibility testing, confirmation of dementia and cognitive status was determined based on multiple measures done by evaluators. A limitation of the study was the sample size and the lack of a gold standard for measurement of pain among older adults living with dementia. All participants were from a single state. Although the MDS pain assessments used were those done closest to when the PAINAD observation was done, timing varied between these measures. Further assessments were done only at a single point in time. We do not collect data on which staff nurse completed the assessments and there was no testing of inter-rater reliability of the assessments. The PAINAD was consistently done during times of some activity, which was not necessarily the case when staff completed the MDS assessments.

## CONCLUSIONS

The findings from this study reinforce the challenges in measurement of pain among older adults living with dementia, support the potential value of using an objective measure of pain particularly for individuals with BIMS scores of 0 to 7, and to consider using both a subjective report of pain and an objective assessment for those with BIMS scores 8 to 12 even when the resident can verbally express their pain. Further, the findings serve as a reminder to not only evaluate pain but to manage it with nonpharmacologic and pharmacologic interventions. Future research on pain assessment in this population should consider why staff do not use observation methods to assess pain in residents with dementia, the timing of treatment and activity relative to assessments, the use of both a verbal and objective measures at the same time for all participants, evaluation of pain at different time points, and inclusion of physiological factors associated with pain.

## Supplementary Material

Tables S1-S5

The following supplementary materials are available online, Table S1: Activities the participant was doing during pain assessment (total N = 156), Table S2: Verbal pain responses among residents based on minimum data base (N = 151), Table S3: Observational pain assessments completed by staff among residents based on minimum data set (N = 26 observed), Table S4: Nonpharmacologic interventions prescribed and used with residents, Table S5: Pain medications prescribed.

## Figures and Tables

**Figure 1.**
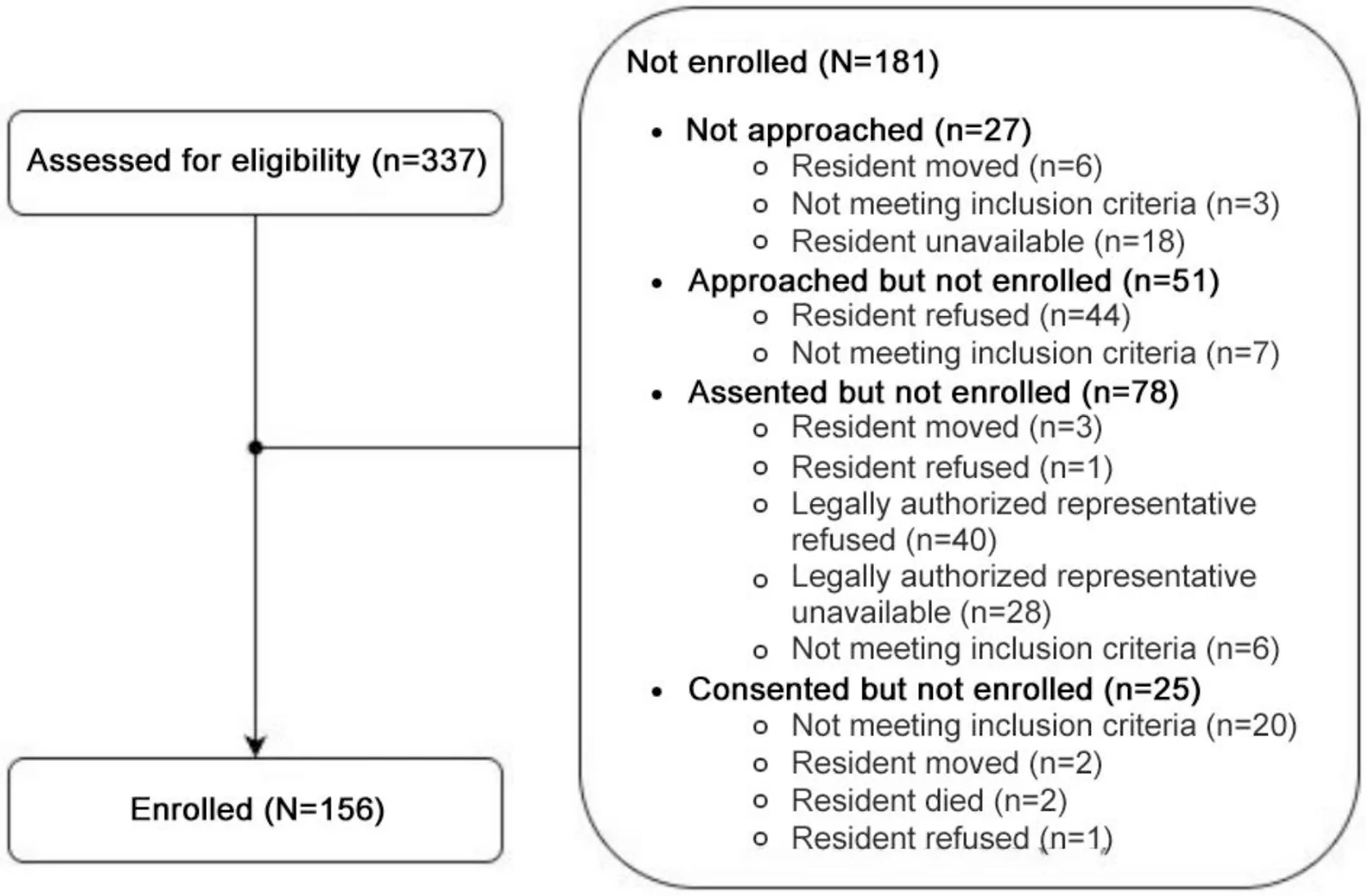
Consort Diagram.

**Table 1.** Minimum Data Set (MDS) Pain Assessment Items (Residents are asked to recall pain over the past 5 days)*.

MDS Item (Section J)	Response (score)	Response (score)			
Have you had pain or hurting at any time	Yes (1)	No (0)			
Has the pain made it hard to sleep	Yes (1)	No (0)			
Have you limited your day-to-day activity due to pain	Yes (1)	No (0)			
How much of the time have you experienced pain or hurting	Almost constantly (4)	Frequently (3)	Occasionally (2)	Rarely (1)	No Answer (0)
Rate your pain from 0 (no pain) −10 (worst pain)					
Describe the intensity of your worst pain	Mild (1)	Moderate (2)	Severe (3)	Very Severe (4)	
Staff assessment of indicators for pain for those who can’t verbally respond	Nonverbal sounds (1)	Vocal complaints of pain (1)	Facial expression of pain (1)	Protective body movement (1)	No pain noted (0)
Provide frequency of complaints or observed signs of pain	1–2 days (1)	3–4 days (2)	Daily (3)		

* Points allocated to each item are noted in ().

**Table 2.** Description of the Sample.

Variable	Mean (Std. Deviation)	Median	N (%)
Sex			
Male			49(31)
Female			107(69)
Race			
White			84(54)
Black/Other			72(46)
Ethnicity			
Hispanic			4(3)
Non-Hispanic			144(92)
Missing			8(5)
Marital Status			
Married			27(17)
Not Married			129(83)
Education			
<High School			8(5)
High School			85(54)
>High School			52(33)
Missing			11(7)
Musculoskeletal Disease			
Yes			53(34)
No			103(66)
Neurological Disease			
Yes			70(45)
No			86(55)
Diabetes			
Yes			82(53)
No			74(47)
Cognition (range 0–13)	5.52(4.04)	5.00	
Age	79.86(9.56)		
Comorbidities (range 0–11)	5.85(2.00)		
Total pain medications ordered	1.21(1.0)		
Total non-pharmacologic interventions ordered	1.8(2.0)		
PAINAD	0.75(1.48)	0.00	114(73)
1			9(6)
2			11(7)
3			12(8)
4			4(3)
5			5(3)
Missing			1(1)
MDS Score Verbal (N = 151)	1.00(0.03)		
No pain			131(87)
Some pain			20(13)
MDS Score Observation (N = 26)	0.50(0.91)		
No pain			19(73)
Some pain			7(27)

**Table 3.** Type of Pain Assessment Used by Staff.

Assessment Type	N (%)
Verbal MDS assessment only	133(86)
Observational MDS assessment only	10(7)
No Staff Assessment Done in MDS	2(1)
Both (verbal and observational)	11(7)
Verbal Assessment Used for those with BIMS ≥ 8 (N = 88 individuals with BIMS ≥ 8)	
Yes	85(98)
No	2(2)
Died	1(1)
Observation Assessment Used for those with BIMS < 8 (N = 66)	
Yes	14(21)
No	52(79)

**Table 4.** Multivariate Analysis for each of the Pain Assessment Tools Used.

Variable	Odds Ratio	Std. err.	Z	P
Minimum Data Set Verbal Measure				
Cognition	1.19	0.08	2.57	0.01
Musculoskeletal Disease	0.92	0.52	−0.14	0.89
Neurological Disease	0.35	0.21	−1.73	0.08
Diabetes	0.90	0.52	−0.18	0.86
Pain medication	1.2	0.29	0.70	0.49
Non-pharmacologic Treatment	0.82	0.14	−1.14	0.26
Age	0.97	0.03	−0.78	0.44
Sex (1 is female; 0 is male)	0.79	0.46	−0.41	0.68
Race (0 is White; 1 is Black)	0.45	0.25	−1.43	0.15
Marital status (0 is not married; 1 is married)	0.48	0.37	−0.96	0.34
Minimal Data Set Observation Measure				
Cognition	0.59	0.20	−1.53	0.13
Musculoskeletal Disease	0.54	0.70	−0.47	0.64
Neurological Disease	0.57	0.87	−0.36	0.72
Diabetes	9.14	18.12	1.12	0.26
Pain medication	4.70	4.03	1.80	0.07
Non-pharmacologic Treatment	0.72	0.34	−0.69	0.49
Observation Measure Using the PAINAD				
Cognition	1.00	0.07	0.10	0.92
Musculoskeletal Disease	6.30	3.55	3.26	0.001
Neurological Disease	0.84	0.45	−0.32	0.75
Diabetes	0.1	0.54	−0.01	0.99
Pain medication	0.63	0.15	−1.89	0.06
Non-pharmacologic Treatment	1.18	0.14	1.38	0.17
Age	0.99	0.03	−0.36	0.72
Sex	1.04	0.59	0.08	0.94
Race	1.04	0.52	0.09	0.93
Marital status	1.17	0.73	0.25	0.80

## Data Availability

Data will be made available upon request of the individual interested to the first author.

## References

[1] Helvik A, Benth JS, Wu B, Engedal K, Selbaek G. Persistent use of psychotropic drugs in nursing home residents in Norway. BMC Geriatr. 2017;17(1):1–15. doi: 10.1186/s12877-017-0440-5 PMC5307887 28193181

[2] Helvik A, Bergh S, Tevik K. A systematic review of prevalence of pain in nursing home residents with dementia. BMC Geriatr. 2023;23(1):641. doi: 10.1186/s12877-023-04340-z PMC10566134 37817061

[3] Cole C, Blackburn J, Carpenter JS, Chen CX, Hickman SE. Pain and associated factors in nursing home residents. Pain Manag Nurs. 2023;24(4):384–92. doi: 10.1016/j.pmn.2023.03.002 PMC10440293 37003932

[4] Centers for Medicare & Medicaid Services. Minimum Data Set 3.0 Public Reports. Available from: https://www.cms.gov/data-research/computer-data-systems/minimum-data-sets-3.0-public-reports. Accessed on 2026 Mar 20.

[5] Monroe TB, Gore JC, Chen LM, Mion LC, Cowan RL. Pain in people with Alzheimer’s disease: Potential applications for psychophysical and neurophysiological research. J Geriatr Psychiatry Neurol. 2012;25(4):240–55. doi: 10.1177/0891988712466457 PMC4455539 23277361

[6] Anderson A, Iversen W, Carter M, Moss K, Cowan R, Monroe T. Experimentally evoked pain in Alzheimer’s disease. J Am Assoc Nurse Pract. 2021;34(1):18–25. doi: 10.1097/JXX.0000000000000580 PMC9118535 33731557

[7] Jesdale B, Bova C, Mbrah A, Lapane K. Self- and staff-reported pain in relation to contextual isolation in long-term nursing home residents with Alzheimer’s disease and related dementias. Geriatr Nurs. 2024;55:161–7. doi: 10.1016/j.gerinurse.2023.11.006 PMC10872894 38000331

[8] Bullock L, Bedson J, Jordan B, Bartlam C, Chew-Graham C, Campbell P. Pain assessment and pain treatment for community-dwelling people with dementia: A systematic review and narrative synthesis. Int J Geriatr Psychiatry. 2019;34(6):807–21. doi: 10.1002/gps.5078 30724409

[9] Pu L, Coppieters MW, Smallbrugge M, Jones C, Byrnes J, Todorovic M, Associations between facial expressions and observational pain in residents with dementia and chronic pain. J Adv Nurs. 2024;80(9):3846–55. doi: 10.1111/jan.16063 38334268

[10] Overen C, Larsson M, Hummelvoll Hillestad A, Karlsson I, Eriksen S. The process of pain assessment in people with dementia living in nursing homes: A scoping review. Palliat Care Soc Pract. 2025;19. doi: 10.1177/26323524241308589 PMC11705334 39776523

[11] Sampson E, Davies N, Vickerstaff V. Evaluation of the psychometric properties of PainCHek in older general hospital patients with dementia. Age Ageing. 2025;54(2):afaf027. doi: 10.1093/ageing/afaf027 PMC11836425 39967417

[12] Yang Q, Yi R, Wang N, Zhang P. Perception, behavior and experience of nursing assistants towards pain of older adults with dementia: A qualitative study. Geriatr Nurs. 2024;56:100–7. doi: 10.1016/j.gerinurse.2024.02.013 38340431

[13] Warden V, Hurley AC, Volicer L. Development and psychometric evaluation of the pain assessment in advanced dementia (PAINAD) Scale. J Am Med Dir Assoc. 2003;4(1):9–15. doi: 10.1097/01.JAM.0000043422.31640.F7 12807591

[14] Resnick B, Boltz M, Galik E, Ellis J, Kuzmik A, Drazich B. Optimal approach for assessing evidence of dementia for research studies. West J Nurs Res. 2023;45(3):253–61. doi: 10.1177/01939459221127587 36380573

[15] Mansbach WE, Mace RA, Clark KM. Differentiating levels of cognitive functioning: a comparison of the Brief Interview for Mental Status (BIMS) and the Brief Cognitive Assessment Tool (BCAT) in a nursing home sample. Aging Ment Health. 2014;18(7):921–8. doi: 10.1080/13607863.2014.899971 24679128

[16] Galvin J, Roe C, Powlishta K, Coats MA, Muich SJ, Grant E, The AD8, a brief informant interview to detect dementia. Neurology. 2005;65(4):559–64. doi: 10.1212/01.wnl.0000172958.95282.2a 16116116

[17] O’Bryant S, Waring S, Cullum C, Hall J, Lacritz L, Massman P, Staging dementia using Clinical Dementia Rating Scale Sum of Boxes Scores. Archives of Neurology, 2008;65(8):1091–5. doi: 10.1001/archneur.65.8.1091 PMC3409562 18695059

[18] Pfeffer R, Kurosaki T, Harrah C, Chance J, Filos S. Functional Activities Questionnaire (FAQ, PFAQ). APA Psycological Tests. Available from: 10.1037/t04022-000. Accessed 2026 Feb 20.

[19] Resnick B, Gruber-Baldini A, Aboff-Petzer I, Galik E, Russ K, Zimmerman S. Reliability and validity of the evaluation to sign consent measure. Gerontologist. 2007;47(1):69–77. 10.1093/geront/47.1.69 17327542

[20] Saliba D, Buchanan J, Edelen MO, Streim J, Ouslander J, Berlowitz D, MDS 3.0: Brief interview for mental status. J Am Med Dir Assoc. 2012;13(7):611–7. 10.1016/j.jamda.2012.06.004 22796362

[21] Cohen-Mansfield J. The relationship between different pain assessments in dementia. Alzheimer Dis Assoc Disord. 2008;22(1):86–93. 10.1097/WAD.0b013e3181630b5c 18317252

[22] Resnick B, Van Haitsma K, Kolanowski J, Galik E, Boltz M, Ellis J, Invariance of the PAINAD scale between the Black and White residents living with dementia. Front Pain Res. 2021;2:757654. doi: 10.3389/fpain.2021.757654 PMC8915686 35295523

[23] DeWaters T, Callahan MF, McCann JJ, Paice JA. Comparison of self-reported pain and the PAINAD scale in hospitalized cognitively impaired and intact older adults after hip fracture surgery. Orthop Nurs. 2008;27(1):21–8. doi: 10.1097/01.NOR.0000310607.62624.74 18300684

[24] Natavio T, McQuillen E, Dietrich MS, Wells N, Rhoten BA, Vallerand AH, A comparison of the Pain Assessment Checklist for Seniors with Limited Ability to Communicate (PACSLAC) and Pain Assessment in Advanced Dementia Scale (PAINAD). Pain Manag Nurs. 2020;21(6):502–9. doi: 10.1016/j.pmn.2020.04.001 32475696

[25] Kawalek C, Dening K. Recognition and assessment of pain in people with advanced dementia. Br J Community Nurs. 2024;29(12):588–95. doi: 10.12968/bjcn.2024.0071 39656038

[26] Centers for Medicare & Medicaid Services. Revised long-term care (LTC) surveyor guidance: Significant revisions to enhance quality and oversight of the LTC survey process. Available from: https://www.cms.gov/medicare/health-safety-standards/quality-safety-oversight-general-information/policy-memos/policy-memos-states-and-cms-locations/revised-revised-long-term-care-ltc-surveyor-guidance-significant-revisions-enhance-quality-and-0. Accessed on 2026 Mar 20.

[27] Centers for Medicare & Medicaid Services. Minimum Data Set (MDS) 3.0 Resident Assessment Instrument (RAI) manual. Available from: https://www.cms.gov/medicare/quality/nursing-home-improvement/resident-assessment-instrument-manual. Accessed on 2026 Mar 20.

[28] Herr K. Pain assessment strategies in older patients. J Pain. 2011;12(3 Suppl 1):S3–13. 10.1016/j.jpain.2010.11.011 21396599

[29] Pu L, Barton M, Gamage MK, Okada M, Todorovic M, Moyle W. Pain assessment and management in dementia care: Qualitative perspectives of people with dementia, their families, and healthcare professionals. J Clin Nurs. 2025;34(8):2933–47. 10.1111/jocn.17771 PMC12181161 40200556

[30] Tsai Y, Browne G, Inder K. Documented nursing practices of pain assessment and management when communicating about pain in dementia care. J Adv Nurs. 2022;78(10):3174–86. 10.1111/jan.15251 PMC9545301 35436007

[31] Parkman S, Mastel-Smith B, McGuire A. Insights to identifying and managing pain in persons with dementia in long-term care: A mixed methods study comparing the Abbey Pain Scale and Pain Assessment in Advanced Dementia Scale. J Gerontol Nurs. 2021;47(8):1–7. 10.3928/00989134-20210113-01 33497447

[32] Liu J, Lai C. Implementation of observational pain management protocol for residents with dementia: A cluster-RCT. J Am Geriatr Soc. 2017;65(3):e56–63. doi: 10.1111/jgs.14763 28152167

[33] Tsai Y, Browne G, Inder K. The effectiveness of interventions to improve pain assessment and management in people living with dementia: A systematic review and meta-analyses. J Adv Nurs. 2021;77(3):1127–40. doi: 10.1111/jan.14660 33222273

[34] Chen Y, Lin L, Watson R. Validating nurses’ and nursing assistants’ report of assessing pain in older people with dementia. J Clin Nurs. 2010;19(1-2):42–52. doi: 10.1111/j.1365-2702.2009.02950.x 20500243

[35] Galik E, Resnick B, Mocci E, Renn C, Song Y, Dorsey S. Differential gene expression in pain-related genes are not affected by the presence of dementia. Pain Manag Nurs. 2024;25(2):145–51. doi: 10.1016/j.pmn.2023.11.001 PMC11016462 38135606

