# Supplementary material for: Pain Assessment in Nursing Home Residents Living with Dementia: Current Findings and Concerns
Source: Adv Geriatr Med Res. Author manuscript; Available in PMC 2026 Nov 7. (PMC13742109; doi:10.20900/agmr20260007)
Supplement: Supplementary file 1 [file NIHMS2217307-supplement-Tables_S1-S5.pdf]

SUPPLEMENTARY MATERIALS

Table S1. Activities the participant was doing during pain assessment (total N = 156).

	Frequency	Percent
Missing	6	3
Range of Motion (active or passive)	96	62
Bathing	5	3
Bed mobility	2	1
Dressing	1	1
Eating	9	6
Lying in bed	2	1
Medication administration	1	1
Marching seated	2	1
Re-positioning	2	1
Sitting in wheelchair	5	3
Standing	4	3
Transferring	4	3
Turning	2	1
Walking	9	6
Wheeling self	5	3
Wound care	1	1

Table S2. Verbal pain responses among residents based on minimum data base (N = 151).

Item	N	%
Have you had any pain or hurting at any time in the last 5 days		
No	131	84%
Yes	20	13%
How much of the time have you experienced pain or hurting		
Don't know	3	2%
Rarely	7	5%
Occasionally	7	5%
Frequently	1	1%
Almost constantly	2	1%
No pain	131	87%
Has the pain made it hard to sleep		
No	17	11%
Yes	3	2%
No pain	131	87%
Have you limited your day-to-day activity due to pain		
No	15	11%
Yes	5	22%
No pain	131	87%
Rate your pain from 0 (no pain) to 10 (worst pain)		
3	2	2%
4	3	2%
5	3	2%
7	4	3%
8	1	2%

10	2	2%
No pain	131	77%
Describe the intensity of your worst pain		
Mild	2	1%
Moderate	3	2%
Not applicable per participant	15	97%
No pain	131	
Total Verbal Rating Score for Pain (range 0–21)		
0	131	87%
1	2	1%
4	1	1%
5	4	3%
7	2	1%
9	3	1%
10	3	2%
11	1	1%
12	1	1%
15	1	1%
16	1	1%

Table S3. Observational pain assessments completed by staff among residents based on minimum data set (N = 26 observed).

**Item**	**N**	**%**
Nonverbal Sounds		
Yes	1	1%
Vocal Complaints of Pain	5	3%
Facial Expressions of Pain	2	1%
Protective Body Movements	1	1%
Total Observation Rating Scale Score for Pain		
0	19	73%
1	2	7%
2	4	15%
3	1	5%

Table S4. Nonpharmacologic interventions prescribed and used with residents.

**Nonpharmacologic Intervention**	**Used by Resident N (%)**
Heat	
Yes	8(5)
No	148(95)
Ice	
Yes	8(5)
No	148(95)
Positioning	
Yes	67(43)
No	89(57)
Ointments	
Yes	30(22)
No	106(78)
Pain patches	
Yes	17(11)
No	139(89)

Tens Devices	
Yes	1(1)
No	155(99)
Physical Activity	
Yes	19(12)
No	137(88)
Massage	
Yes	10(6)
No	146(94)
Distraction	
Yes	16(10)
No	140(90)
Cognitive Behavioral Therapy	
Yes	0(0)
No	156(100)
Pet Visits	
Yes	2(2)
No	154(98)
Relaxation Techniques	
Yes	17(11)
No	139(89)
Acupuncture/Acupressure	
Yes	0 (0)
No	136(100)
Deep Breathing	
Yes	1(1)
No	155(99)
Aromatherapy	
Yes	0 (0)
No	156(100)
Music Therapy	
Yes	14(9)
No	142(91)
Biofeedback	
Yes	0 (0)
No	156(100)
Hypnosis	
Yes	0 (0)
No	156(100)
Prescribed Exercises from Rehabilitation	
Yes	18(12)
No	138(88)
Passive Range of Motion	
Yes	12(7)
No	144(93)
Active Range of Motion	
Yes	13(8)
No	143(92)
Other Interventions<br>Guided imagery; Activities; Pressure Relieving Mattress; Reading; Television; Snacks	
Yes	10(6)
No	166(94)

Table S5. Pain medications prescribed.

Pain Medication	Prescribed N	% of Sample
Acetaminophen	104	67%
Aspercream	10	6%
Cyclobenzaprine	1	1%
Lidocaine Patch	21	14%
Gabapentin	22	14%
Hydrocodone	3	2%
Morphine	9	6%
Oxycodone	6	4%
Pregabalin	3	2%
Tramadol	6	4%
Indomethacin or Ibuprofen	2	2%
Tylenol with Codeine	1	1%
